# Do sociodemographic variables and cardiometabolic risk factors moderate the mere-measurement effect on physical activity and sedentary time?

**DOI:** 10.1186/s12872-020-01551-9

**Published:** 2020-06-05

**Authors:** Lisa Voigt, Antje Ullrich, Sophie Baumann, Marcus Dörr, Ulrich John, Sabina Ulbricht

**Affiliations:** 1 Institute for Community Medicine, Department of Social Medicine and Prevention, University Medicine Greifswald, Walther-Rathenau-Str. 48, 17475 Greifswald, Germany; 2 DZHK (German Centre for Cardiovascular Research), partner site Greifswald, Greifswald, Germany; 3 Institute and Policlinic of Occupational and Social Medicine, Faculty of Medicine, Technische Universität Dresden, Dresden, Germany; 4 Department of Internal Medicine B, University Medicine Greifswald, Greifswald, Germany

**Keywords:** Mere-measurement effect, Question-behavior effect, Reactivity, Cardiometabolic risk factors, Physical activity, Sedentary time

## Abstract

**Background:**

Participation in an assessment may change health behavior. This “mere-measurement effect” may be used for prevention purposes. However, little is known about whether individuals’ characteristics moderate the effect. The objective was to explore whether changes of physical activity (PA) and sedentary time (ST) after a cardiovascular assessment depend on sociodemographic variables and cardiometabolic risk factors.

**Methods:**

A sample of *n* = 175 adults aged 40 to 65 received baseline assessment including self-administered PA and ST questionnaires and standardized measurement of blood pressure, waist circumference, and blood parameters. After 5 weeks, participants again reported PA and ST without any prior treatment or intervention. Linear regression models were used to analyze the dependence of five-week changes in PA and ST on baseline sociodemographic and cardiometabolic variables.

**Results:**

Men increased transport-related PA more than women (*b* = 9.3 MET-hours/week, *P* = .031). Men with higher triglycerides increased transport-related PA less than men with lower triglycerides (*b* = − 5.6 MET-hours/week, *P* = .043). Men with higher systolic blood pressure reduced ST more than those with lower systolic blood pressure (*b* = − 35.7 min/week, *P* = .028). However, this linear association ceased to exist at a level of approximately 145 mmHg (*b* of squared association = 1.0, *P* = .080). A similar relationship was found for glycated hemoglobin and ST.

**Conclusions:**

The findings suggest that sex and cardiometabolic risk factors moderate mere-measurement effects on PA and ST. Researchers and practitioners using mere measurement for prevention purposes may address PA and ST according to these individual characteristics.

**Trial registration:**

ClinicalTrials.govNCT02990039. Registered 7 December 2016. Retrospectively registered.

## Background

Participation in an assessment may change the behavior that is aimed to be investigated [1, 2]. In health behavior research, such effects have been called “mere-measurement effect”, “assessment reactivity”, or “question-behavior effect” [3, 4]. A meta-analysis found small but significant effect sizes for measurement of physical activity (PA) [4]. Altering PA can occur as a result of wearing a device [5, 6] or filling out a questionnaire on past behavior or on cognitions related to PA [7, 8]. Several mechanisms underlying the mere-measurement effect have been discussed. Participants may change their behavior, for example, as a result of reflecting on their attitudes or on discrepancies between beliefs and actual behavior [9].

It has been suggested that the mere-measurement effect could be used as a simple and cost-effective intervention to improve health behavior across a wide range of people [8, 10–12]. Thus, it needs to be verified that the potential benefit of the effect is not systematically attenuated among groups of individuals according to sociodemographic and health related characteristics. However, there is a lack of evidence about sex, age, and socioeconomic status as moderators of the mere-measurement effect. A recent study on several health behaviors could not conclusively demonstrate a difference in the effect across socioeconomic groups which would potentially lead to increased health inequalities [13].

Associations between regular PA and cardiovascular health are well established [14–16]. Evidence for increased health risks of prolonged sedentary time (ST) is accumulating [17, 18]. Thus, it seems of particular interest whether the mere-measurement effect on PA and ST differentially affects individuals with various cardiometabolic risk factors. If improvements of PA and ST result from an increased awareness of discrepancies between desired and actual behavior, individuals with a less favorable cardiometabolic risk profile may be more likely to respond to the mere-measurement effect than individuals with a more favorable risk profile. In contrast, if general benefits from the effect are less pronounced among those with a less favorable cardiometabolic risk profile, health promotion using mere measurement may fail to reach those with the higher need.

In a previous study, it was found that participants of a cardiovascular examination program subsequently increased PA for transport and tended to decrease ST without any formal treatment, referral, or intervention [19]. As little research has been conducted on individual characteristics of the participants, the objective of the present study was to explore whether the mere-measurement effect on leisure-time PA (PA_leisure_), transport-related PA (PA_transport_), and ST after attending a cardiovascular examination is moderated by sociodemographic variables (sex, age, and employment) and cardiometabolic risk factors (systolic blood pressure [SBP], waist circumference, glycated hemoglobin [HbA1c], total cholesterol, high-density lipoprotein [HDL], and triglycerides).

## Methods

### Study sample

**Fig. 1:**
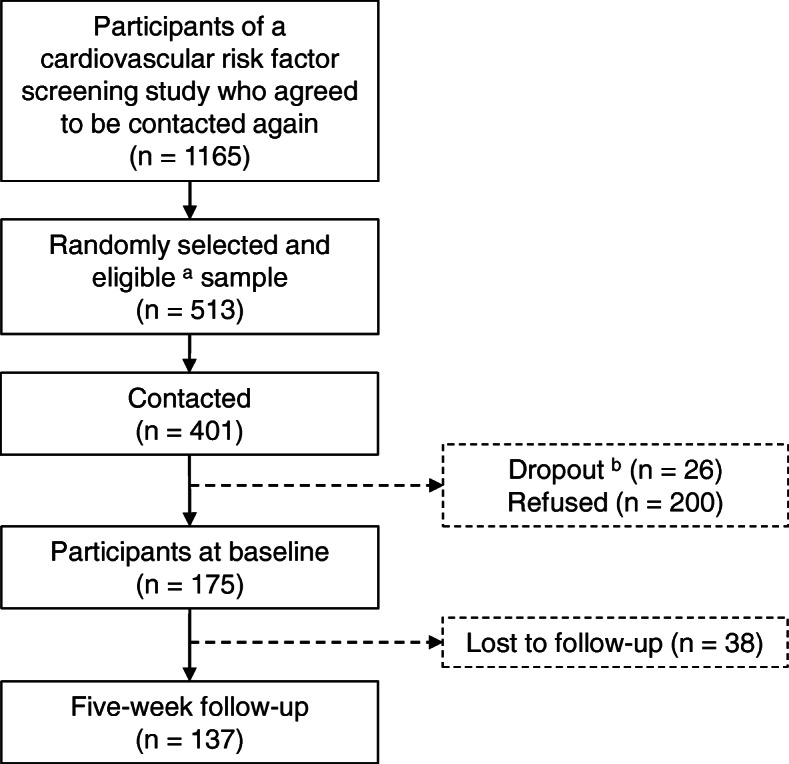
Flow of participation. ^a^ Eligibility criteria: age ≥ 40 and ≤ 65 years, no history of cardiovascular event (myocardial infarction or stroke) or vascular intervention, self-reported body mass index ≤35 kg/m^2^, resident in a pre-defined zip-code area. ^b^ not in age range, had died, had a cardiovascular event or intervention, were too ill to participate, or moved away

Participants of this study were recruited for a cardiovascular risk factor screening study at general practices, job centers, and via one statutory health insurance company in Northeastern Germany between June 2012 and December 2013. The study is described more detailed elsewhere [20]. Among individuals who agreed to be contacted again (*n* = 1165, 95%) 513 persons were randomly selected who fulfilled the following eligibility criteria: age ≥ 40 and ≤ 65 years, no history of cardiovascular event (myocardial infarction or stroke) or vascular intervention, self-reported body mass index ≤35 kg/m^2^, and residency in a pre-defined zip-code area. Of those, 401 individuals were offered participation in a study aimed to assess the feasibility of a tailored counselling letter intervention to increase PA and to reduce ST during leisure time. A number of 175 agreed and gave written informed consent for participation. The study was conducted between February 2015 and August 2016. For the present analyses, the data from baseline assessment (*n* = 175) and first follow-up assessment (*n* = 137; 78%) of the feasibility study were used to explore the mere-measurement effect. The follow-up was conducted 5 weeks after baseline (Fig. 1).

### Procedure

All participants were invited via letter to the cardiovascular examination center of the University Medicine Greifswald for baseline assessment. Participants completed self-administered paper-pencil questionnaires on PA, ST, and sociodemographic variables and received blood sample taking as well as standardized measurement of blood pressure, waist circumference, body height, and body weight. The follow-up assessment was realized 5 weeks after baseline and comprised only a self-administered paper-pencil questionnaire on PA and ST sent via letter mail. The study was approved by the clinical ethical committee of the University Medicine Greifswald (protocol number BB 002/15a).

### Measures

#### Physical activity and sedentary time

To assess PA and ST at baseline and follow-up, the long form of the International Physical Activity Questionnaire (IPAQ) was used [21]. The IPAQ is a widely used questionnaire with very good test-retest reliability and acceptable validity for adults aged 18 to 65. Frequency, duration, and intensity of PA during the last seven days are measured in four domains of life including leisure time and transportation. PA_leisure_ comprises walking, PA on a moderate-intensity level, and PA on a vigorous-intensity level. PA_transport_ includes walking and cycling. In order to sum time spent in each domain, time spent on one activity is multiplied by its metabolic equivalent of task (MET) value, which accounts for the intensity of the activity. ST is reported separately for weekdays and weekend days and independently of the domain. PA_leisure_ and PA_transport_ in MET-hours per week and (combined weekday and weekend) ST in minutes per week were calculated according to the IPAQ protocol [22].

#### Sociodemographics

Sex, age (years), employment (yes/no), and current living together with a partner (yes/no) was assessed at baseline by a self-administered questionnaire.

#### Cardiometabolic risk factors

Blood pressure was assessed at the cardiovascular examination center in a seating position via standardized measurement using a digital blood pressure monitor (705IT, Omron Corporation, Tokyo, Japan). After a five-minute resting period, blood pressure was measured three times with 3 min rest between each measurement by trained and certificated medical staff. For data analysis, the mean of second and third measurement of systolic and diastolic blood pressure (mmHg) were used. Antihypertensive medication prescribed within the last 12 months (yes/no) was assessed by questionnaire. Waist circumference (cm) was measured midway between lowest rib and iliac crest using an inelastic tape. Non-fasting blood samples were taken and HbA1c (mmol/mol), plasma total cholesterol (mmol/L), HDL (mmol/L), and triglycerides (mmol/L) were determined by standard methodology at the Institute for Clinical Chemistry and Laboratory Medicine of the University Medicine Greifswald.

### Statistical analysis

Data were analyzed with Stata/SE version 14.2 [23]. Multiple imputation using chained equations was performed to account for missing data. The approach of this method is to use the distribution of the observed data to estimate a set of plausible values for the missing data. The proportion of incomplete cases was 44, 37, and 32% for analyses on PA_leisure_, PA_transport_, and ST, respectively. 80 imputed data sets were used, which were combined to obtain the overall estimates, variances, and confidence intervals for linear regression models of PA_leisure_, PA_transport_, and ST. The imputation model was built using the outcomes, predictors, and covariates of the main analysis models. In addition, a number of seven auxiliary variables (diastolic blood pressure, body height, school education, partnership, duration of night sleep, TV time, and transportation in motor vehicles) were included to improve the imputation model. To account for skewed continuous variables, the predictive mean matching method was used [24].

Three outcomes were investigated: five-week differences of self-reported PA_leisure_, PA_transport_, and ST (calculated as follow-up value minus baseline value). Linear regression analyses were calculated to estimate associations between sociodemographic characteristics as well as cardiometabolic risk factors and the outcomes. Robust standard errors were used to account for potential estimation bias. First, associations between sex, age, and employment and the outcomes were investigated in one model separately for each outcome. Employment but not school education as an indicator for socioeconomic status was investigated because non-participation in this study was associated with lower education and the number of individuals with < 10 years of school education was low (*n* = 12). Because of sex-specific thresholds of some cardiometabolic risk factors (e.g., waist circumference) analyses across all three outcomes were stratified by sex. In addition, the stratification is justified as sex differences were found for PA_transport_ (results presented in supplementary table S1 (see Additional file 1)). Second, each cardiometabolic risk factor was tested in a separate model. All models were adjusted for age, employment, duration between baseline and follow-up, and baseline PA_leisure_, PA_transport_, or ST, respectively. Associations between SBP and the outcomes were additionally adjusted for blood pressure lowering medication. In all analyses, likelihood ratio tests were used to decide whether to include quadratic terms of age or cardiometabolic risk factors in the models. *P*-values < .05 were considered statistically significant. As the study was not powered for subgroup analyses, findings with *P*-values < .10 are reported additionally indicating trends towards moderation effects. In addition to the main analysis using multiply imputed data, a sensitivity analysis was conducted using complete cases.

## Results

### Sample characteristics

There were *n* = 112 women (64.0%) and *n* = 63 men (36.0%) in the study sample. The mean age was 54.4 years (SD = 6.2) and 80.8% were employed. The mean difference of PA_leisure_ between baseline and follow-up was *M* = 4.3 MET-hours per week (SD = 29.0) and, accordingly, *M* = 6.5 MET-hours per week (SD = 20.3) for PA_transport_ and *M* = − 163.2 min per week (SD = 1039.5) for ST. The mean duration between baseline and follow-up was 39.2 days (SD = 8.9). Descriptive statistics of cardiometabolic risk factors are shown in Table 1.

**Table 1:** Characteristics of the study sample (*n* = 175)

Variables	Overall (*n* = 175)		Women (*n* = 112)		Men (*n* = 63)		*P*-Value
	n	Values	n	Values	n	Values	
**Sociodemographic variables**							
Age (years)	175	54.4 ± 6.2	112	54.6 ± 6.2	63	54.0 ± 6.1	ns
Employment (yes)	172	139 (80.8%)	110	88 (80.0%)	62	51 (82.3%)	ns
**Cardiometabolic risk factors**							
Systolic blood pressure (mmHg)	172	126.8 ± 14.7	109	123.0 ± 13.9	63	133.4 ± 13.7	<.001
Diastolic blood pressure (mmHg)	172	76.6 ± 9.5	109	75.1 ± 9.0	63	79.1 ± 9.9	.009
Blood pressure lowering medication (yes)	167	67 (40.1%)	104	47 (45.2%)	63	20 (31.8%)	ns
Waist circumference (cm)	173	91.6 ± 12.5	110	87.9 ± 12.3	63	98.1 ± 10.0	<.001
HbA1c (mmol/mol)	170	39.1 ± 6.0	108	39.3 ± 6.5	62	38.9 ± 4.9	ns
Total cholesterol (mmol/L)	171	5.3 ± 1.0	109	5.4 ± 1.1	62	5.0 ± 0.9	.034
HDL (mmol/L)	168	1.4 ± 0.4	106	1.5 ± 0.4	62	1.2 ± 0.3	<.001
Triglycerides (mmol/L)	172	1.6 ± 1.0	111	1.5 ± 0.9	61	1.9 ± 1.1	.008
**Context variables**							
Duration from baseline to follow-up (days)	137	39.2 ± 8.9	90	38.6 ± 6.7	47	40.4 ± 12.1	ns
**Physical activity and sedentary time**							
Baseline leisure-time physical activity (MET-hours/week)	141	22.1 ± 28.7	88	21.1 ± 25.2	53	23.9 ± 34.1	ns
Baseline transport-related physical activity (MET-hours/week)	149	17.7 ± 23.2	95	17.8 ± 22.6	54	17.5 ± 24.6	ns
Baseline sedentary time (minutes/week)	158	2538.2 ± 1146.2	101	2604.6 ± 1103.7	57	2420.5 ± 1219.0	ns
Difference in leisure-time physical activity (MET-hours/week)	98	4.3 ± 29.0^a^	61	2.7 ± 25.4^a^	37	6.9 ± 34.3^a^	ns
Difference in transport-related physical activity (MET-hours/week)	110	6.5 ± 20.3^a^	72	2.0 ± 16.4^a^	38	15.0 ± 24.3^a^	.001
Difference in sedentary time (minutes/week)	120	−163.2 ± 1039.5^a^	79	−175.9 ± 929.4^a^	41	−138.7 ± 1236.8^a^	ns

Data are presented as mean ± standard deviation for continuous variables and as the number of participants (%) for categorical variables. Presented *P*-values for comparisons between women and men are based on t-test for continuous variables and chi-square test for categorical variables

*HbA1c* glycated hemoglobin, *HDL* high-density lipoprotein, *MET* metabolic equivalent of task, *ns* not significant

^a^ Positive mean values indicate an increase from baseline to follow-up and negative values indicate a reduction

### Associations between sociodemographic characteristics and physical activity and sedentary time

Men increased PA_transport_ more than women (*b* = 9.3 MET-hours/week [95% CI: 0.9; 17.7], *P* = .031) and older individuals tended to increase PA_transport_ more than younger individuals (*b* = 0.5 MET-hours/week [95% CI: − 0.03; 1.1], *P* = .065, Table S1). After stratification by sex, the association between PA_transport_ and age disappeared. There was a tendency towards a quadratic association between age and the reduction of ST in women (linear term: *b* = 0.8 min/week [95% CI: − 34.0; 35.5], *P* = .965; quadratic term: *b* = − 6.5 [95% CI: − 13.4; 0.3], *P* = .061). No associations between employment and the outcomes were found (Table 2).

**Table 2:** Results of linear regression analyses for sociodemographic characteristics separately for women (*n* = 112) and men (*n* = 63)

	Leisure-time physical activity ∆ (MET-hours per week)		Transport-related physical activity ∆ (MET-hours per week)		Sedentary time ∆ (minutes per week)	
	Women	Men	Women	Men	Women	Men
	*b* [95% CI]	*b* [95% CI]	*b* [95% CI]	*b* [95% CI]	*b* [95% CI]	*b* [95% CI]
Age (years)	0.7 [−0.3; 1.7]	0.5 [− 0.9; 1.8]	0.5 [− 0.2; 1.2]	0.8 [− 0.3; 1.9]	0.8 [−34.0; 35.5]	− 10.8 [−68.1; 46.5]
Age squared	–	–	–	–	− 6.5 [− 13.4; 0.3]^+^	–
Employment (Ref. yes)	3.8 [− 12.5; 20.0]	13.6 [−18.8; 45.9]	1.7 [−9.7; 13.1]	−2.1 [− 25.1; 20.8]	−81.6 [− 583.6; 420.4]	421.2 [− 374.6; 1217.1]

Five-week changes are calculated as follow-up value minus baseline value. Results are based on multiply imputed data. Coefficients were adjusted for all other variables shown in the table, duration to follow-up, and baseline value of leisure-time physical activity, transport-related physical activity, or sedentary time, respectively

∆ Five-week change, *MET* metabolic equivalent of task, *b* unstandardized regression coefficient, *CI* confidence interval; − not included

^+^*P <* .10; based on robust standard errors

### Associations between cardiometabolic risk factors and physical activity and sedentary time

**Fig. 2:**
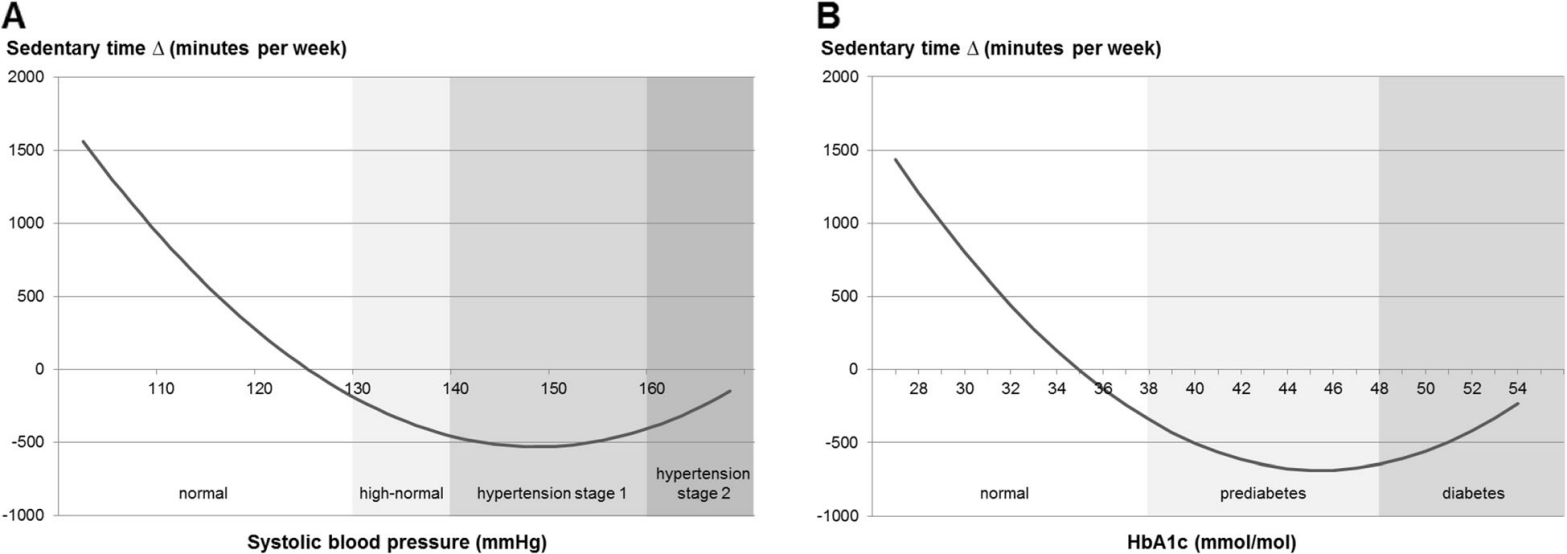
Five-week changes of sedentary time in men dependent on systolic blood pressure (**a**) and HbA1c (**b**). *N* = 63. Results were adjusted for age, employment, duration to follow-up, and baseline value of sedentary time. Coefficients of systolic blood pressure were additionally adjusted for blood pressure lowering medication

In men, results indicated a U-shaped association between SBP and the reduction of ST (linear term: *b* = − 35.7 min/week [95% CI: − 67.3; − 4.0], *P* = .028; quadratic term: *b* = 1.0 [95% CI: − 0.1; 2.1], *P* = .080; Table 3, Fig. 2a). In women, there was a trend towards a quadratic association between SBP and the increase of PA_leisure_ (linear term: *b* = 0.2 MET-hours/week [95% CI: − 0.2; 0.7], *P* = .306; quadratic term: *b* = 0.02 [95% CI: − 0.003; 0.04], *P* = .093). In men, a U-shaped association between HbA1c and the reduction of ST (linear term: − 93.0 min/week [95% CI: − 152.6; − 33.4], *P* = .003; quadratic term: *b* = 6.2 [95% CI: − 0.4; 12.9], *P* = .064; Fig. 2b) was found.

**Table 3:** Results of linear regression analyses for cardiometabolic risk factors separately for women (n = 112) and men (n = 63)

	Leisure-time physical activity ∆ (MET-hours per week)		Transport-related physical activity ∆ (MET-hours per week)		Sedentary time ∆ (minutes per week)	
	Women	Men	Women	Men	Women	Men
	*b* [95% CI]	*b* [95% CI]	*b* [95% CI]	*b* [95% CI]	*b* [95% CI]^a^	*b* [95% CI]
SBP (mmHg)^b^	0.2 [−0.2; 0.7]	0.01 [−0.5; 0.5]	0.04 [− 0.3; 0.3]	0.3 [− 0.4; 1.0]	−7.4 [−21.5; 6.7]	− 35.7 [−67.3; −4.0]*
SBP squared^b^	0.02 [− 0.003; 0.04]^+^	–	–	–	–	1.0 [− 0.1; 2.1]^+^
Waist circumference (cm)	−0.1 [− 0.6; 0.5]	0.3 [− 0.5; 1.1]	0.003 [− 0.3; 0.3]	0.4 [− 0.3; 1.1]	0.7 [− 16.1; 17.4]	−11.7 [−36.9; 13.4]
HbA1c (mmol/mol)	0.6 [− 0.4; 1.5]	1.2 [− 0.6; 3.1]	−1.1 [− 0.6; 0.4]	0.7 [− 1.0; 2.4]	−16.2 [− 58.9; 26.5]	−93.0 [− 152.6; −33.4]**
HbA1c squared	–	–	–	–	–	6.2 [− 0.4; 12.9]^+^
Total cholesterol (mmol/L)	2.6 [−2.9; 8.1]	− 0.6 [− 13.4; 12.1]	1.0 [− 2.5; 4.4]	−5.2 [− 13.6; 3.2]	−70.8 [− 259.8; 118.2]	−24.8 [− 413.9; 364.2]
HDL (mmol/L)	3.9 [− 9.7; 17.5]	−4.2 [− 31.8; 23.4]	10.6 [−1.2; 22.4]^+^	3.2 [− 20.5; 26.8]	−18.8 [− 565.2; 527.6]	−562.8 [− 1590.4; 464.9]
HDL squared	–	–	–	–	–	− 1928.3 [− 3912.8; 56.2]^+^
Triglycerides (mmol/L)	−2.1 [− 7.8; 3.6]	−1.0 [− 8.8; 6.8]	−1.6 [− 5.0; 1.7]	−5.6 [− 11.1; − 0.2]*	− 12.9 [− 224.8; 199.0]	−35.6 [− 326.6; 255.4]

Five-week changes are calculated as follow-up value minus baseline value. Results are based on multiply imputed data. Coefficients were adjusted for age, employment, duration to follow-up, and baseline value of leisure-time physical activity, transport-related physical activity, or sedentary time, respectively

∆ Five-week change, *MET* metabolic equivalent of task, *b* unstandardized regression coefficient, *CI* confidence interval, *SBP* systolic blood pressure, *HbA1c* glycated hemoglobin, *HDL* high-density lipoprotein; − not included

^+^*P* < .10, *P < .05, **P < .01; based on robust standard errors

^a^ Coefficients were additionally adjusted for age squared as indicated by likelihood ratio test

^b^ Coefficients were additionally adjusted for blood pressure lowering medication

In men, there was a trend towards a quadratic association between HDL and the reduction of ST (linear term: *b* = − 562.8 min/week [95% CI: − 1590.4; 464.9], *P* = .275; quadratic term: *b* = − 1928.3 [95% CI: − 3912.8; 56.2], *P* = .057). Women with higher HDL tended to increase PA_transport_ more than women with lower HDL (*b* = 10.6 MET-hours/week [95% CI: − 1.2; 22.4], *P* = .077). Men with higher triglycerides increased PA_transport_ less than men with lower triglycerides (*b* = − 5.6 MET-hours/week [95% CI: − 11.1; − 0.2], *P* = .043). No associations between waist circumference or total cholesterol and the outcomes were found. Sensitivity analyses using complete cases yielded similar results, which are presented in supplementary tables S2, S3, and S4 (see Additional file 1).

## Discussion

This study aimed to explore moderators of the mere-measurement effect in adults as indicated by associations between sociodemographic variables and cardiometabolic risk factors and changes in PA and ST after attending a cardiovascular examination program. First, men increased PA_transport_ more than women. Age tended to be positively associated with PA_transport_ improvements. Second, among men, results revealed U-shaped associations both between SBP and HbA1c and the reduction of ST. And, men with higher triglycerides increased PA_transport_ less than men with lower triglycerides.

Responding to an assessment on PA or ST may trigger new thinking about the behavior. Within focus group discussions, some participants of this study reported that after completing the questionnaire they were negatively surprised by the amount of time they spent sedentarily. This may explain the relatively high proportions of participants who have changed their behavior in a positive way. Improvements between baseline and follow-up in PA_leisure,_ PA_transport,_ and ST were reported by 48, 56, and 52% of the participants, respectively.

There was an increase in PA_transport_ of 9.3 MET-hours per week in men as compared to women. To put this value into perspective, the IPAQ assigns 3.3 METs to walking and 6.0 METs to cycling [22]. This means that men spent, for example, an additional 3 h walking or 1.5 h cycling per week compared to women. This difference may result from a worse health condition among men as indicated by less favorable values of SBP, HDL, and triglycerides. In line with suggested mechanisms underlying the mere-measurement effect [3, 9], men might have altered their behavior as their awareness of the relationship between behavior and health increased in response to reflecting on activity levels when completing a detailed 27-item-questionnaire combined with the assessment of cardiometabolic risk factors. Further, evidence suggests that the built environment is an important factor associated with active transport [25, 26]. Changing PA_transport_ may not be achievable to any individual due to long distances between home and work or other daily responsibilities that require transportation using motor vehicles.

Despite the fact that the present sample was restricted to 40- to 65-year olds, it was found that older participants tended to increase PA_transport_ more than younger participants. This may be contrary to expectations as prior meta-analyses comparing student samples with non-student samples including older adults hint at a larger measurement effect among young adults compared to older adults [9, 27]. However, compared to younger participants, older participants in this sample had higher levels of HbA1c, total cholesterol, and triglycerides (data not shown). Thus, older participants had a worse health condition and, therefore, may have been more motivated to increase PA.

Associations between SBP as well as HbA1c and ST in men indicate that those with less favorable risk factors improve more than those with more favorable risk factors. In contrast, results on PA point to the opposite direction. Associations between triglycerides and PA_transport_ in men and between HDL and PA_transport_ in women revealed that those with more favorable values improved their behavior more than those with less favorable values. Thinking about weekly ST independent of PA levels might have motivated men with a less favorable risk profile to alter inactivity on a lower threshold, in contrast to men in good health, who altered PA rather than ST. Nevertheless, the U-shaped associations indicate that men with blood pressure levels beyond 150 mmHg and men with HbA1c values on the threshold to diabetes seem to refrain from ST reductions possibly due to their worse physical condition. Compared to men, women in this sample had more favorable cardiometabolic risk factors which may explain why these factors did not moderate changes of PA and ST in women.

The present study was conducted to shed some light on a topic that is still a niche area in clinical research. However, there are four limitations to consider. First, a selection of highly motivated individuals is likely. The proportion of individuals who declined participation was high (53%) and non-participation was associated with smoking, lower education, and female sex. Thus, the findings may not be generalizable to the general population. Second, systematic changes in PA and ST observed in this non-controlled study do not necessarily imply mere-measurement effects. To reduce potential confounding, adjustments were made for variables related to individuals’ characteristics and data collection. Future research on transport-related PA should take additional context variables into account, e.g., the distance between home and work. Third, PA and ST were assessed using self-report measures. Due to social desirability bias [28, 29], an over-reporting of PA or an under-reporting of ST might have occurred in this study. Recent research revealed higher odds of having metabolic syndrome for men who did not meet PA guidelines according to accelerometry data than for men who met the guidelines [30]. However, this relationship disappeared when PA was measured via self-report, which seems to hint at an over-reporting of PA by men with metabolic syndrome. Similarly, in this sample, men with less favorable risk factors might have under-reported ST. Future studies could assess behavior change via direct measures, e.g. accelerometry, using wearing periods of at least 2 weeks since prior research suggested the presence of reactivity bias during the first week of measurement [5, 6]. Finally, the findings may suffer from a lack of power to detect differences between subgroups, as this study was not particularly designed to investigate moderators of the mere-measurement effect.

## Conclusion

The findings of this study suggest that beneficial alterations of PA and ST after a cardiovascular examination program may be moderated by sex, age, and cardiometabolic risk factors. Researchers and practitioners using the mere-measurement effect to promote behavior change should consider these individual characteristics. For example, completing a questionnaire on PA or ST while waiting in a physician’s practice may trigger new thinking about a behavior in a patient. If cardiometabolic risk factors are assessed, a deeper awareness of the relationship between inactivity and health risks may be raised. In the course of this, men with a less favorable risk profile, for example, may be more responsive to answering a questionnaire on ST instead of PA. Future research using larger sample sizes is needed to verify the moderators found in this exploratory study and to investigate long-term effects on behavior and health.

## Supplementary information

**Additional file 1: **Supplementary results. **Table S1.** Results of linear regression analyses regarding associations between sociodemographic characteristics and changes in self-reported physical activity and sedentary time. **Tables S2-S4.** Results of sensitivity analyses using complete cases.

## Data Availability

The data that support the findings of this study are available from the corresponding author on reasonable request. Researchers requesting the data will be required to sign a contract ensuring data usage in compliance with the statement given in the informed consent procedure and with the German data protection law, that the data will not be transferred to others, and that the data will be deleted after the intended analysis has been completed. The data are not publicly available due to potential privacy restrictions. To comply with the statement given in the informed consent procedure, the use of the data is restricted to medical research purposes. We cannot ensure to prevent use for other purposes when uploading the data for public access.

## Abbreviations

HbA1c: Glycated hemoglobin
HDL: High-density lipoprotein
IPAQ: International physical activity questionnaire
MET: Metabolic equivalent of task
PA: Physical activity
PA_leisure_: Leisure-time physical activity
PA_transport_: Transport-related physical activity
SBP: Systolic blood pressure
ST: Sedentary time
