# Supplementary material for: Do sociodemographic variables and cardiometabolic risk factors moderate the mere-measurement effect on physical activity and sedentary time?
Source: BMC Cardiovasc Disord. 2020 Jun 5;20:272. doi: 10.1186/s12872-020-01551-9 (PMC7275363; doi:10.1186/s12872-020-01551-9)
Supplement: Supplementary file 1 — Additional file 1: Supplementary results. Table S1. Results of linear regression analyses regarding associations between sociodemographic characteristics and changes in self-reported physical activity and sedentary time. Tables S2-S4. Results of sensitivity analyses using complete cases. [file 12872_2020_1551_MOESM1_ESM.docx]

**Additional file 1**

**Supplementary results**

**Manuscript Title: Do Sociodemographic Variables and Cardiometabolic Risk Factors Moderate the Mere-Measurement Effect on Physical Activity and Sedentary Time?**

Table S1. Results of linear regression analyses regarding associations between sociodemographic characteristics and changes in self-reported physical activity and sedentary time (*n* = 175)

|  | Leisure-time  physical activity ∆  (MET-hours per week) |  | Transport-related  physical activity ∆  (MET-hours per week) |  | Sedentary time ∆  (minutes per week) |
| --- | --- | --- | --- | --- | --- |
|  | *b* [95% CI] |  | *b* [95% CI] |  | *b* [95% CI] |
| Sex (Ref. women) | 3.1 [-7.3; 13.5] |  | 9.3 [0.9; 17.7]* |  | -16.3 [-386.5; 353.9] |
| Age (years) | 0.6 [-0.2; 1.4] |  | 0.5 [-0.0; 1.1]^+^ |  | -4.2 [-35.0; 26.7] |
| Employment (Ref. yes) | 6.9 [-8.8; 22.5] |  | 0.7 [-10.3; 11.7] |  | 2.4 [-434.3; 439.0] |

∆ Five-week change; MET: metabolic equivalent of task; b: unstandardized regression coefficient; CI: confidence interval

Five-week changes are calculated as follow-up value minus baseline value. Results are based on multiply imputed data. Coefficients were adjusted for all other variables shown in the table, duration to follow-up, and baseline value of leisure-time physical activity, transport-related physical activity, or sedentary time, respectively.

^+^ P < .10, * P < .05; based on robust standard errors

Table S2. Results of linear regression analyses regarding associations between sociodemographic characteristics and changes in self-reported leisure-time physical activity (*n* = 98), transport-related physical activity (*n* = 110), and sedentary time (*n* = 119) using complete cases

|  | Leisure-time  physical activity ∆  (MET-hours per week) |  | Transport-related  physical activity ∆  (MET-hours per week) |  | Sedentary time ∆  (minutes per week) |
| --- | --- | --- | --- | --- | --- |
|  | *b* [95% CI] |  | *b* [95% CI] |  | *b* [95% CI] |
| Sex (Ref. women) | 5.8 [-5.7; 17.2] |  | 12.9 [3.8; 22.0]** |  | -82.1 [-462.9; 298.7] |
| Age (years) | 0.5 [-0.5; 1.5] |  | 0.3 [-0.3; 0.8] |  | -0.9 [-34.5; 32.8] |
| Age squared | - |  | -0.1 [-0.2; -0.0]** |  | - |
| Employment (Ref. yes) | 5.5 [-14.7; 25.7] |  | 2.5 [-9.5; 14.4] |  | 125.8 [-206.7; 458.3] |

∆ Five-week change; MET: metabolic equivalent of task; b: unstandardized regression coefficient; CI: confidence interval; - not included

Five-week changes are calculated as follow-up value minus baseline value. Coefficients were adjusted for all other variables shown in the table, duration to follow-up, and baseline value of leisure-time physical activity, transport-related physical activity, or sedentary time, respectively.

** P < .01; based on robust standard errors

Table S3. Results of linear regression analyses regarding associations between sociodemographic characteristics and changes in self-reported leisure-time physical activity (*n* = 98), transport-related physical activity (*n* = 110), and overall sedentary time (*n* = 119) separately for women and men using complete cases

|  | Leisure-time physical activity ∆  (MET-hours per week) | | |  | Transport-related physical activity ∆  (MET-hours per week) | | |  | Sedentary time ∆  (minutes per week) | | |
| --- | --- | --- | --- | --- | --- | --- | --- | --- | --- | --- | --- |
|  | Women |  | Men |  | Women |  | Men |  | Women |  | Men |
|  | *b* [95% CI] |  | *b* [95% CI] |  | *b* [95% CI] |  | *b* [95% CI] |  | *b* [95% CI] |  | *b* [95% CI] |
| Age (years) | 0.4 [-1.0; 1.7] |  | 0.5 [-0.8; 1.9] |  | 0.3 [-0.3; 0.9] |  | 0.8 [-0.4; 2.0] |  | 11.9 [-22.3; 46.0] |  | -20.8 [-75.5; 34.0] |
| Age squared | - |  | - |  | -0.1 [-0.2; -0.0]* |  | - |  | -11.4 [-18.3; -4.5]** |  | - |
| Employment (Ref. yes) | -1.0 [-15.2; 13.3] |  | 18.7 [-32.3; 69.7] |  | 4.1 [-7.4; 15.7] |  | -10.3 [-34.9; 14.3] |  | 35.6 [-329.2; 400.3] |  | 824.5 [290.0; 1359.0]** |

∆ Five-week change; MET: metabolic equivalent of task; b: unstandardized regression coefficient; CI: confidence interval; - not included

Five-week changes are calculated as follow-up value minus baseline value. Coefficients were adjusted for all other variables shown in the table, duration to follow-up, and baseline value of leisure-time physical activity, transport-related physical activity, or sedentary time, respectively.

* P < .05, ** P < .01; based on robust standard errors

Table S4. Results of linear regression analyses regarding associations between cardiometabolic risk factors and changes in self-reported leisure-time physical activity (*n* = 98), transport-related physical activity (*n* = 110), and sedentary time (*n* = 119) using complete cases

|  | Leisure-time physical activity ∆  (MET-hours per week) | | |  | Transport-related physical activity ∆  (MET-hours per week) | | |  | Sedentary time ∆  (minutes per week) | | |
| --- | --- | --- | --- | --- | --- | --- | --- | --- | --- | --- | --- |
|  | Women |  | Men |  | Women |  | Men |  | Women |  | Men |
|  | *b* [95% CI] |  | *b* [95% CI] |  | *b* [95% CI] |  | *b* [95% CI] |  | *b* [95% CI] ^a^ |  | *b* [95% CI] |
| SBP (mm Hg) ^b^ | -0.1 [-0.6; 0.5] |  | -0.1 [-0.7; 0.5] |  | 0.0 [-0.3; 0.9] |  | 0.4 [-0.5; 1.2] |  | -3.9 [-17.8; 9.9] |  | -43.5 [-77.8; -9.3]* |
| SBP squared ^b^ | - |  | - |  | - |  | - |  | - |  | 1.2 [0.1; 2.3]* |
| Waist circumference (cm) | -0.2 [-0.9; 0.5] |  | 0.3 [-0.5; 1.2] |  | -0.1 [-0.4; 0.2] ^a^ |  | 0.3 [-0.4; 1.1] |  | 3.3 [-12.4; 19.0] |  | -15.2 [-38.1; 7.6] |
| HbA1c (mmol/mol) | 0.7 [0.3; 1.2]** |  | 1.7 [-0.5; 3.8] |  | 0.0 [-0.3; 0.4] ^a^ |  | 0.8 [-1.2; 2.9] |  | -3.4 [-56.0; 49.1] |  | -56.5 [-114.4; 1.4]^+^ |
| HbA1c squared | - |  | - |  | - |  | - |  | - |  | 13.6 [8.7; 18.4]*** |
| Total cholesterol (mmol/L) | 2.1 [-4.0; 8.2] |  | -2.2 [-21.4; 17.1] |  | 0.9 [-2.5; 4.3] |  | -7.9 [-15.5; -0.3]* |  | -77.8 [-274.5; 119.0] |  | 20.8 [-503.6; 545.3] |
| HDL (mmol/L) | 3.9 [-9.1; 16.8] |  | -11.2 [-39.0; 16.6] |  | 18.9 [5.4; 32.4]** ^a^ |  | -10.3 [-50.0; 29.3] |  | 17.9 [-582.1; 617.9] |  | -576.9 [-1881.8; 727.9] |
| HDL squared | - |  | - |  | - |  | - |  | - |  | -2553.3 [-5038.7; -67.9]* |
| Triglycerides (mmol/L) | -4.0 [-10.3; 2.3] |  | -2.1 [-13.3; 9.0] |  | -2.1 [-5.2; 0.9] ^a^ |  | -9.9 [-17.2; 2.6]* |  | -21.2 [-212.5; 170.1] |  | -60.1 [-389.7; 269.6] |

∆ Five-week change; MET: metabolic equivalent of task; b: unstandardized regression coefficient; CI: confidence interval; SBP: systolic blood pressure; HbA1c: glycated hemoglobin; HDL: high-density lipoprotein; - not included

Five-week changes are calculated as follow-up value minus baseline value. Coefficients were adjusted for age, employment, duration to follow-up, and baseline value of leisure-time physical activity, transport-related physical activity, or sedentary time, respectively.

^+^ P < .10, * P < .05, ** P < .01, *** P < .001; based on robust standard errors

^a^ coefficients were additionally adjusted for age squared as indicated by likelihood ratio test

^b^ coefficients were additionally adjusted for blood pressure lowering medication
